# Frontal cortical activation during emotional and non-emotional verbal fluency tests

**DOI:** 10.1038/s41598-022-12559-w

**Published:** 2022-05-19

**Authors:** Michael K. Yeung

**Affiliations:** 1 Department of Rehabilitation Sciences, The Hong Kong Polytechnic University, Hong Kong, China; 2 University Research Facility in Behavioral and Systems Neuroscience, The Hong Kong Polytechnic University, Hong Kong, China

**Keywords:** Neuroscience, Psychology

## Abstract

There has been growing recognition of the utility of combining the verbal fluency test and functional near-infrared spectroscopy (fNIRS) to assess brain functioning and to screen for psychiatric disorders. Recently, an emotional analogue of the semantic fluency test (SFT) has been developed that taps partly different processes from conventional verbal fluency tests. Nevertheless, neural processing during the emotional SFT remains elusive. Here, fNIRS was used to compare frontal cortical activation during emotional and non-emotional SFTs. The goal was to determine whether the emotional SFT activated overlapping yet distinct frontal cortical regions compared with the conventional, non-emotional SFT. Forty-three healthy young adults performed the emotional and non-emotional SFTs while hemodynamic changes in the bilateral frontopolar, dorsomedial, dorsolateral, ventrolateral, and posterolateral frontal cortices were measured by fNIRS. There were significant increases in oxyhemoglobin concentration and significant decreases in deoxyhemoglobin concentration (i.e., activation) in frontopolar, dorsolateral, and ventrolateral frontal regions during both the non-emotional and emotional SFTs. Also, complementary analyses conducted on changes in the two chromophores using classical and Bayesian hypothesis testing suggested that comparable frontal cortical regions were activated while performing the two tests. This similarity in activation occurred in a context where non-emotional and emotional SFT performances exhibited differential relationships with the overall level of negative mood symptoms. In conclusion, frontal cortical activation during the emotional SFT is similar to that during the conventional, non-emotional SFT. Given that there is evidence for discriminant validity for the emotional SFT, the neural mechanisms underlying the uniqueness of this test warrant further investigation.

## Introduction

The verbal fluency test is a popular neuropsychological test for language and executive function^1^. This test takes two major forms, requiring the production of as many unique words as possible that either begin with a certain letter (i.e., phonemic fluency test) or belong to a specific semantic category (i.e., semantic fluency test; SFT) within a time limit (e.g., 60 s). Various language and executive function skills are involved during the verbal fluency test. Specifically, it requires accessing lexical-semantic information, switching between concepts or categories, monitoring working memory representations, and inhibiting intrusive and repetitive thoughts^2–4^. Empirical research has supported the clinical utility of the verbal fluency test because this test, particularly the SFT, is sensitive to the effects of aging and many neuropsychiatric disorders, including depression and schizophrenia^5–7^.

Over the last two decades, the verbal fluency test has been increasingly used with functional near-infrared spectroscopy (fNIRS) to study brain functioning and to screen for neuropsychiatric disorders^8,9^. Essentially, fNIRS measures neural activation in terms of increases in oxyhemoglobin concentration (HbO) and decreases in deoxyhemoglobin concentration (HbR) in the cerebral bloodstream^10^. Although this method offers a lower spatial resolution and a shallower measurement depth than functional magnetic resonance imaging (fMRI), fNIRS is more resilient to movement and enables brain activity measurement in a more natural environment^11^. For these reasons, fNIRS is attracting growing attention among researchers. In keeping with the fMRI literature^12^, fNIRS studies have observed activation mainly in the lateral prefrontal cortex (PFC), frontopolar cortex, and anterior temporal cortex during verbal fluency test performance^13–15^. In addition, an fNIRS meta-analysis revealed consistent frontotemporal hypoactivation, regardless of whether test performance was impaired, across neuropsychiatric disorders, including depression and schizophrenia^9^. The clinical utility of the verbal fluency test–fNIRS paradigm is thus supported.

Substantial evidence suggests that emotion and neutral words are processed and retrieved differently^16–18^. Recently, an emotional analogue of the SFT has been developed that requires the production of as many emotion words as possible within a given time^19,20^. The emotional SFT emphasizes the controlled retrieval of emotion lexical content; therefore, it may be useful for studying neuropsychiatric populations that exhibit difficulty in identifying and verbalizing their emotional experiences, including depression, schizophrenia, and autism spectrum disorder^21–23^. While performances on the emotional and non-emotional SFTs are positively correlated^19,24^, they exhibit different relationships with other cognitive tests and psychophysiological measures. Specifically, the level of physiological arousal in terms of heart rate and the frequency of skin conductance response at time of task positively correlated with emotional but not animal fluency performance^19^. Also, performance on the emotional SFT, but not the phonemic or semantic verbal fluency tests, positively correlated with the ability to understand and interpret the emotions and intentions of other people^20^. In contrast, performance on the phonemic and semantic verbal fluency tests, but not the emotional SFT, positively correlated with language proficiency skills^25^. Therefore, preliminary data suggest that the emotional SFT captures partly different processes than the conventional verbal fluency tests.

While the neural substrates of conventional verbal fluency tests are quite well understood, those of the emotional SFT remain elusive. This dearth of knowledge limits the full use of the emotional SFT itself and the verbal fluency test–fNIRS method. Within the frontal lobes, various regions and associated circuits specialize in different functions. Specifically, the left ventrolateral PFC, via its connection with the anterior temporal cortex, is involved in the controlled access to memory^26^, and the right ventrolateral PFC interacts with the supplementary motor cortex to inhibit responses^27^. The dorsolateral PFC, via its connection with the lateral parietal cortex, allows for shifting attention between conceptual sets^28^ and for monitoring working memory representations^29,30^. In addition, the medial PFC, via its connection with the amygdala, plays a dominant role in emotion processing^31,32^. The frontopolar cortex is specialized in the integration and coordination of (abstract) information through interconnections with the supramodal PFC^33,34^.

Given that both the emotional and non-emotional SFTs require the controlled retrieval of lexical-semantic information, they may similarly engage the lateral PFC. Also, since the two tests differ in the involvement of emotion processing, they may activate the medial PFC differently. Here, fNIRS was used to compare frontal cortical activation during the emotional and non-emotional SFTs. The goal was to determine whether the emotional SFT activated overlapping yet distinct frontal cortical regions compared with the conventional, non-emotional SFT. For the emotional SFT, participants generated either positive or negative emotion words. For the non-emotional SFT, they produced either country or occupation names^35^. Although animal is the most commonly used category for the non-emotional SFT, considering the possible differences between concrete and abstract word processing^36^, abstract categories were employed here to facilitate comparison with the emotional SFT. Nevertheless, the animal fluency test was also administered after fNIRS recording as a validation task. Also, since a negative mood has been shown to be associated with an enhanced processing of emotion words, regardless of the valence^18^, the relationship between negative mood symptoms and emotional SFT performance was also investigated.

## Methods

### Participants

Fifty-one Chinese young adults aged 18–39 years were recruited via poster advertisement on the campus of the Hong Kong Polytechnic University. Exclusion criteria, which were based on self-report, included: (1) a history of any psychiatric or neurological disorder, (2) stroke or traumatic brain injury that required hospitalization, (3) currently taking any psychotropic medication, (4) non-fluent Cantonese speaking, (5) left-handedness as determined by the short form of the Edinburgh Handedness Inventory (EHI-SF)^37^. All participants self-reported normal or corrected-to-normal vision.

Eight participants were subsequently excluded for the following reasons: reporting discomfort during the fNIRS assessment (*n* = 4), speaking non-fluent Cantonese (*n* = 1), being left-handed (*n* = 1; mean EHI-SF score = − 100), and having at least one missing fNIRS channel cluster due to excessive bad channels (*n* = 2). Thus, the analytic sample consisted of 43 young adults (19 males, 24 females) aged 18–37 years (*M* = 25.4, *SD* = 5.6). The included and excluded individuals were statistically comparable in age and sex distribution (*t*-test and Fisher’s exact test: *p*s > 0.88). Written informed consent was obtained from each participant prior to the experiment. This study was approved by the Human Subjects Ethnics sub-committee at the Hong Kong Polytechnic University (HSEARS20201110006) and conducted in accordance with the Declaration of Helsinki.

### Procedure and materials

Eligible individuals were invited to participate in a fNIRS study that took place at the University Research Facility in Behavioral and Systems Neuroscience at the Hong Kong Polytechnic University. Participants were asked to abstain from caffeine and alcohol intake on the day of the experiment. After obtaining written informed consent, the participants performed the SFTs in the context of fNIRS recording in a quiet, dimly lit room.

**Figure 1:**
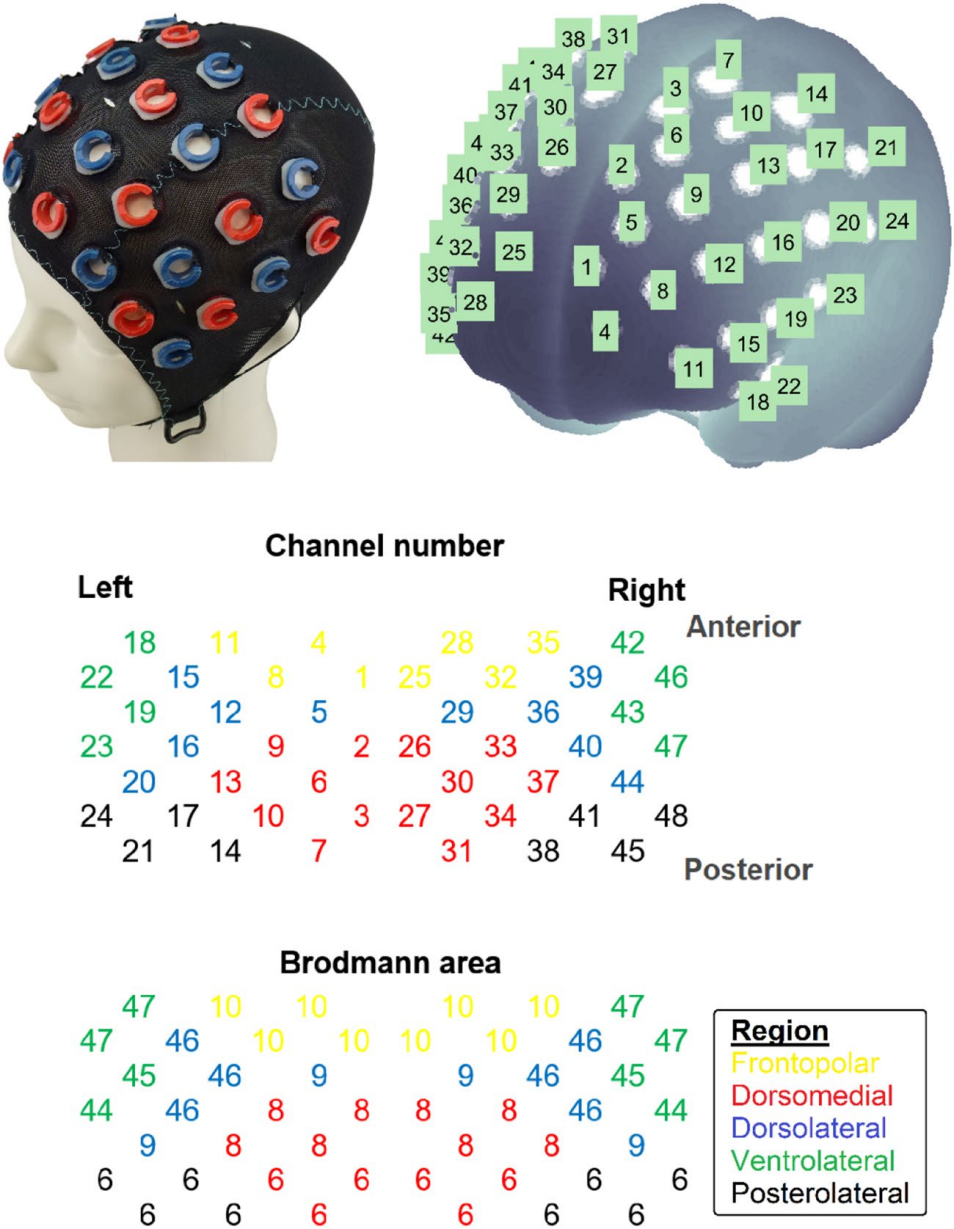
Channel Arrangement and Clustering. Participants wore an EasyCap mounted with 16 sources and 16 detectors. These optodes were arranged in a 4 × 8 matrix, centering at Fz overall. Based on the probes’ coordinates in the 10–20 system, the neuroanatomical locations of the 48 measurement channels were estimated. Channels were divided into five frontal lobe subregions, based on Brodmann areas.

The SFT paradigm was adapted from previous fNIRS studies (Fig. 1)^14,15,38,39^. Participants were asked to generate as many unique words as possible from a specific semantic category within 60 s for four abstract categories (country, occupation, positive emotion, negative emotion). The order of presentation was randomized and different for each individual. The production of country and occupation names constituted the conventional, non-emotional SFT, whereas the generation of positive and negative emotion words represented the emotional SFT.

Participants performed a control task for 30 s at the beginning of the trial and for 60 s following each 60-s block of the semantic fluency task. For the control task, we instructed participants to repeat the phrase “1, 2, 3, 4” at the pace of one digit per second. The purpose of the control task was to account for changes in fNIRS signals due to oral and facial muscle movement. The entire task lasted 510 s. Participants sat approximately 70 cm away from a computer screen. They were asked to perform tasks according to the cues shown at the center of the screen. The cues indicated either the category for word production or the repetition phrase. The cue stimuli were presented on a 17-inch Dell monitor with a 5:4 aspect ratio using E-Prime 3.0 (Psychology Software Tools, Pittsburgh, PA). Before the experimental task began, we asked participants to sit still and minimize head movement throughout the task. They were given an abstract category example (i.e., surname) to get familiar with the task.

After fNIRS recording, participants completed the animal fluency task, which required the production of as many animal names as possible within 60 s. In addition, the 21-item version of the Chinese Depression Anxiety Stress Scales (DASS-21) was administered to measure the level of negative mood symptoms over the last week^40,41^. This questionnaire asked participants to rate 21 statements on a four-point Likert scale, ranging from 0 (never) to 3 (almost always). A higher score implied greater symptoms. The total DASS-21 score that represented the overall level of negative mood symptoms was analyzed.

### fNIRS measurement

A 48-channel ETG-4000 system (Hitachi Medical Co., Tokyo, Japan) was used to measure hemodynamic changes across the frontal cortex during the SFTs. The machine used 695 and 830 nm lights and sampled data at a rate of 10 Hz. Participants wore an EasyCap adjusted to their head size that was mounted with 16 emitters and 16 detectors (Fig. 1). The emitters and detectors were alternatingly positioned and arranged in two 4 × 4 arrays (i.e., equivalent to a 4 × 8 matrix), centering at Fz overall. Depending on the head circumference, the optode separation varied between 29–31 mm (30 mm for a 56 cm head size) to achieve fixed locations with respect to the 10–20 positions.

Based on the probes’ coordinates in the 10–20 system, the fNIRS probe and channel positions were rendered onto the Montreal Neurological Institute (MNI) standard brain using the NFRI toolbox^42^. The Brodmann area (BA) atlas was then used to label the probabilistic anatomical locations of channels. Based on the highest probabilistic value of the neural structure underneath each fNIRS channel, the 48 channels were divided into five subregions, including the frontopolar (BA 10), dorsomedial (BA 6, 8), dorsolateral (BA 9, 46), ventrolateral (BA 44, 45, 47), and posterolateral (BA 6) frontal cortices. Data were analyzed at the cluster rather than the channel level because the test–retest reliability is higher for the cluster approach^43^. Two participants were excluded for having all bad channels in at least one region (see next section for the definition of bad channels).

### fNIRS data preprocessing

The HomER3 package and custom scripts on MATLAB R2020a (The MathWorks, Inc., Natick, MA) were used to preprocess the fNIRS data^44^. First, channels with an overall signal-to-noise ratio < 20 dB (noisy channels) or > 65 dB (saturated channels) were rejected using the *hmrR_PruneChannels* function, which was modified to exclude channels with an exceptionally high signal-to-noise ratio typical of saturated channels^45^. A mean of 3.5% channels (*SD* = 3.4%) were rejected. Negative values in intensity were then corrected by adding an offset with the aid of the *hmrR_PreprocessingIntensity_Negative* function, followed by converting the raw intensity signals to optical density changes using the *hmrR_Intensity2OD* function.

The temporal derivative distribution repair (TDDR) algorithm, recently shown to be superior to five other motion correction methods, was applied to remove baseline shift and spike artifacts through the *hmrMotionCorrectTDDR* function^46^. Also, principal component analysis was performed using the *hmrR_PCAFilter* function to remove systemic confounds. The first component, which almost always shows maximal correlation with the global average signal^47^, was removed for all participants. Next, a 0.005–0.5 Hz Butterworth bandpass filter was applied to remove slow drifts and cardiac artifacts using the *hmrR_BandpassFilt* function.

The optical density data were then converted to HbO and HbR changes via the modified Beer–Lambert law implemented in the *hmrR_OD2Conc* function. The function was modified to correct the differential pathlength factor for wavelength and age based on the general equation^48^. The *hmrR_BlockAvg* function was then applied to extract the time course of fNIRS measurements for each category, from 10 s before the task onset to 50 s after the task offset. Baseline correction was done using the 10 s before each trial. Lastly, the data were averaged across the two categories for the non-emotion and emotion conditions separately, as well as across all channels (excluding bad channels) for each region. Both HbO and HbR were analyzed to give a complete picture of cerebral hemodynamics.

### Data analysis

The recorded verbal responses on the SFTs were coded by two native Cantonese speakers, and the number of correct and unique words produced was generated for each category before averaging for the non-emotion and emotion conditions separately. Two-way random effects, absolute agreement intraclass correlation coefficients (ICCs) were calculated to evaluate the level of interrater agreement. The interrater agreement is poor for ICCs < 0.40, fair for ICCs between 0.40 and 0.59, good for ICCs between 0.60 and 0.74, and excellent for ICCs ≥ 0.75^49^.

As the Shapiro*–*Wilk tests yielded nonsignificant (*p*s > 0.05) results for all task performance and questionnaire variables (i.e., number of words produced and the DASS-21 total score) and most fNIRS variables (i.e., mean HbO and HbR changes), parametric tests were used for the statistical analyses. For behavioral data, a paired *t*-test was conducted to compare the non-emotional and emotional SFTs. For fNIRS data, like previous studies that compared different verbal fluency test versions (i.e., phonemic vs. semantic)^15^, one-sample *t*-tests were first conducted to determine whether mean frontal HbO and HbR during the non-emotional and emotional SFTs differed from zero. False-discovery-rate correction, which is commonly used in fNIRS research, was applied^50^. Two linear mixed models with subject as a random factor and condition (non-emotion, emotion), hemisphere (left, right), and region (frontopolar, dorsomedial, dorsolateral, ventrolateral, posterolateral), as well as the corresponding two- and three-way interaction terms as predictor variables, were then conducted on the mean changes in HbO and HbR separately. Linear mixed models were solved by Restricted Maximum Likelihood (REML), and the degrees of freedom were calculated using the Satterthwaite approximation^51^. The Sidak correction was used in the post-hoc tests.

Linear mixed models tested whether the null hypothesis could be rejected but did not state evidence for the null hypothesis. In contrast, Bayesian inference quantified evidence for both the null and the alternative hypothesis and provided different information from null hypothesis significance testing^52^. Thus, to supplement information about the activation differences between the two SFT conditions, which were of interest, the Bayes factors were computed to evaluate the ratio of likelihood of the null hypothesis (i.e., no difference between conditions) to the likelihood of the alternative hypothesis (i.e., a difference between conditions)^53,54^. Bayes factor calculation is currently available for simple contrasts, as in paired *t*-tests, but not for linear mixed model effects. As such, the Bayes factors representing the difference in the mean change in HbO or HbR between conditions were calculated for each individual region. According to Jeffreys^53^, a Bayes factor of 1/3 to 3 indicates evidence that is not worth more than a bare mention for either hypothesis. A Bayes factor of 3 to 10 implies substantial evidence in favor of the null hypothesis, and a Bayes factor of 1/10 to 1/3 represents substantial evidence in favor of the alternative hypothesis.

Lastly, Pearson’s correlation analyses were conducted to correlate emotional SFT performance with animal fluency performance in order to validate the emotional SFT, and with the DASS-21 total score to investigate the role of negative mood symptoms in emotion word production. Similar to previous studies^55,56^, bivariate outliers with a Cook’s distance > 0.5 were excluded to reduce the influence of outliers. In addition, these correlations were compared with the correlations observed for the non-emotional SFT using *Z* tests to determine the specificity of the relationships found for the emotional SFT^57^. Statistical analyses were performed using IBM SPSS Statistics for Windows, Version 26.0 (IBM Corp., Armonk, NY). All statistical tests were two-tailed, and the alpha level was set at 0.05.

## Results

### Behavioral results

The total numbers of correct and unique words produced on the non-emotional and emotional SFTs and their interrater reliability estimates are displayed in Table 1. For both versions, all the single-measures and average-measures ICCs were at least 0.97, indicating excellent interrater agreement. Participants produced significantly fewer words on the emotional than the non-emotional SFT, *t*(42) = 18.52, *p* < 0.001, *d* = 2.82.

**Table 1:** Number of Correct and Unique Words Generated on the Semantic Fluency Tests.

	Mean (SD)	Intraclass correlation coefficient (ICC)	
		Single measures [95% CI]	Average measures [95% CI]
**During fNIRS**			
Non-emotion (country + occupation words)	36.0 (7.4)	.979 [.963, .989]	.990 [.981, .994]
Emotion (positive + negative emotion words)	17.2 (5.8)	.991 [.982, .996]	.996 [.991, .998]
**After fNIRS**			
Animal names	23.4 (5.1)	.993 [.987, .996]	.996 [.993, .998]

*CI* confidence interval, *fNIRS* functional near-infrared spectroscopy. Two-way random effects, absolute agreement ICCs are shown.

### Frontal cortical activation

**Figure 2:**
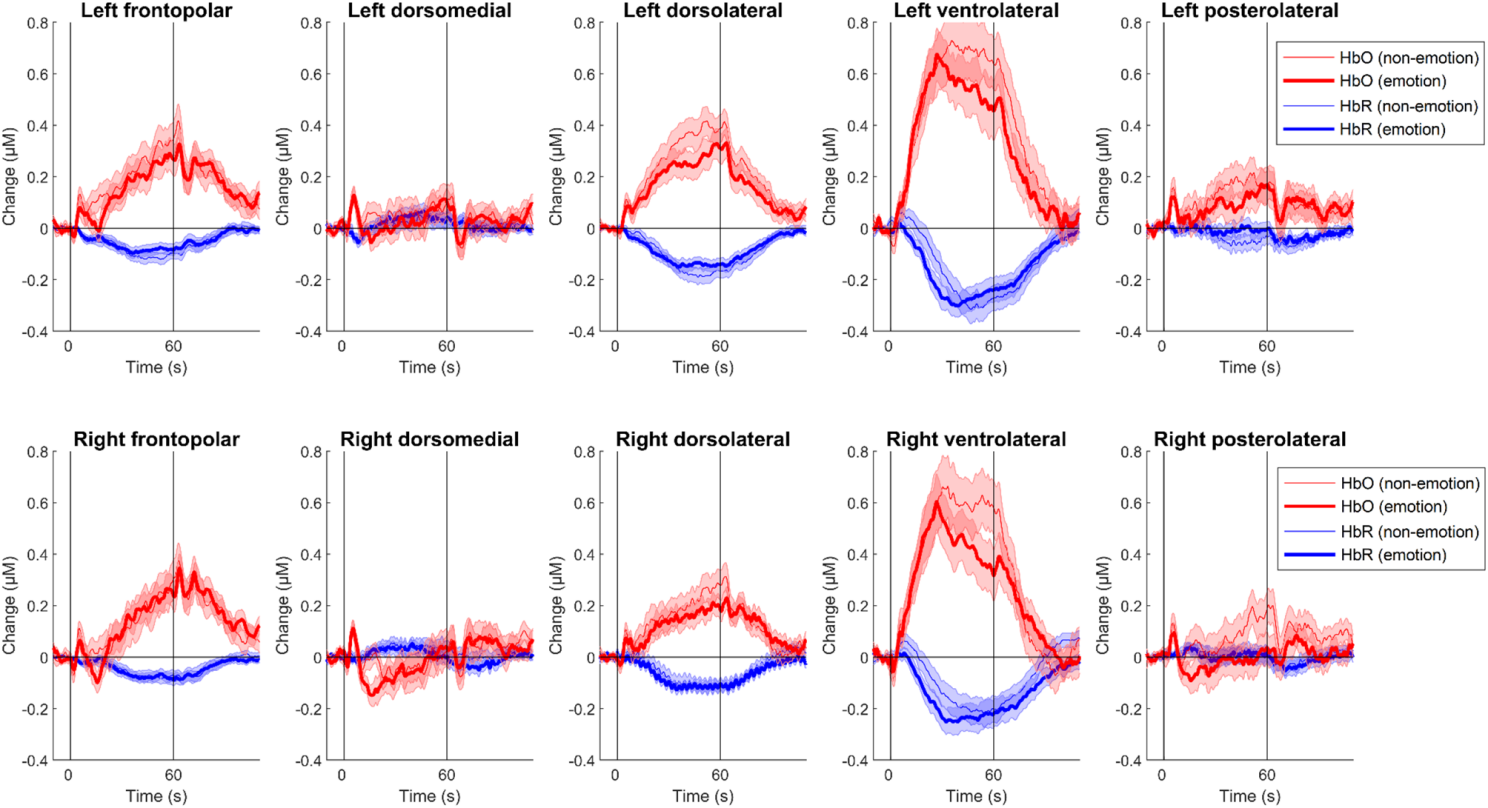
Changes in Oxyhemoglobin (HbO) and Deoxyhemoglobin (HbR) Concentration Over the Time Courses of the Semantic Fluency Tests. Red and blue lines indicate changes in HbO and HbR, respectively. Thin and thick lines represent the non-emotional and emotional semantic fluency tests, respectively. The verbal fluency task started at *t* = 0 s and ended at *t* = 60 s. Error bars denote one standard error ± the mean.

Changes in frontal HbO and HbR over the time courses of the SFTs are illustrated in Fig. 2, and mean changes in the two chromophores are shown in Table 2. First, one-sample *t*-tests with false-discovery-rate corrections were conducted to determine whether there were significant changes in mean HbO and HbR during the SFTs. For the non-emotional SFT, significant HbO increases were observed in the left frontopolar and posterolateral frontal cortex and the bilateral dorsolateral and ventrolateral PFC, *t*s > 3.11, *p*s < 0.003, *d*s from 0.47 to 0.97. These changes were accompanied by significant HbR decreases in the bilateral frontopolar, dorsolateral, and ventrolateral PFC, *t*s > 2.79, *p*s < 0.008, *d*s from 0.43 to 0.91. For the emotional SFT, significant HbO increases were detected in the bilateral frontopolar, dorsolateral, and ventrolateral PFC, *t*s > 2.54, *p*s < 0.015, *d*s from 0.39 to 0.94. These changes were paralleled by significant HbR decreases in the same bilateral regions, *t*s > 3.66, *p*s < 0.001, *d*s from 0.55 to 0.98.

**Table 2:** Mean changes in frontal oxyhemoglobin (ΔHbO) and deoxyhemoglobin (ΔHbR) concentration during the semantic fluency tests.

	Non-emotion			Emotion		
	Mean (*SD*)	*t*	*p*	Mean (*SD*)	*t*	*p*
**ΔHbO (μM)**						
Left						
Frontopolar	0.184 (0.300)	4.04	< .001***	0.140 (0.276)	3.32	.002**
Dorsomedial	0.052 (0.232)	1.48	.15	0.027 (0.249)	0.72	.48
Dorsolateral	0.259 (0.266)	6.36	< .001***	0.196 (0.248)	5.19	< .001***
Ventrolateral	0.510 (0.555)	6.03	< .001***	0.455 (0.485)	6.15	< .001***
Posterolateral	0.128 (0.268)	3.11	.003**	0.084 (0.276)	1.99	.053
Right						
Frontopolar	0.110 (0.352)	2.04	.047	0.101 (0.261)	2.54	.015*
Dorsomedial	− 0.023 (0.223)	− 0.68	.50	− 0.044 (0.303)	− 0.95	.35
Dorsolateral	0.156 (0.274)	3.74	< .001***	0.119 (0.214)	3.65	< .001***
Ventrolateral	0.462 (0.571)	5.31	< .001***	0.371 (0.528)	4.61	< .001***
Posterolateral	0.068 (0.327)	1.36	.18	− 0.018 (0.305)	− 0.38	.71
**ΔHbR (μM)**						
Left						
Frontopolar	− 0.075 (0.098)	− 5.06	< .001***	− 0.063 (0.101)	− 4.05	< .001***
Dorsomedial	0.013 (0.128)	0.65	.52	0.022 (0.153)	0.93	.36
Dorsolateral	− 0.107 (0.118)	− 5.95	< .001***	− 0.101 (0.103)	− 6.47	< .001***
Ventrolateral	− 0.144 (0.236)	− 4.00	< .001***	− 0.183 (0.215)	− 5.58	< .001***
Posterolateral	− 0.027 (0.129)	− 1.36	.18	− 0.006 (0.149)	− 0.25	.80
Right						
Frontopolar	− 0.047 (0.091)	− 3.38	.002**	− 0.049 (0.089)	− 3.66	< .001***
Dorsomedial	0.030 (0.129)	1.53	.13	0.018 (0.123)	0.96	.34
Dorsolateral	− 0.060 (0.127)	− 3.08	.004**	− 0.077 (0.084)	− 5.99	< .001***
Ventrolateral	− 0.103 (0.242)	− 2.79	.008**	− 0.159 (0.248)	− 4.20	< .001***
Posterolateral	0.012 (0.116)	0.69	.49	0.010 (0.139)	0.46	.65

False-discovery-rate correction was applied. *p < .05, **p < .01, ***p < .001.

### Effects of condition, hemisphere, and region on frontal cortical activation

**Figure 3:**
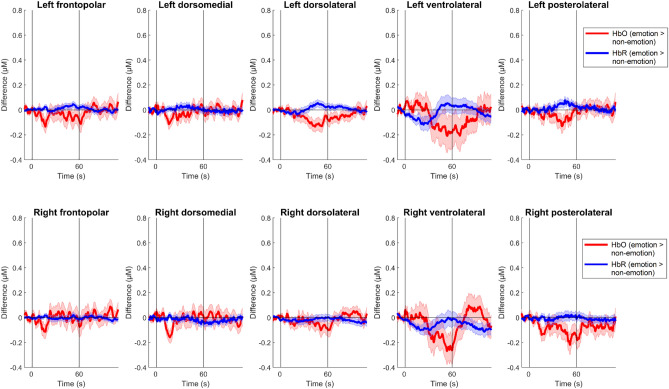
Differences in Oxyhemoglobin (HbO) and Deoxyhemoglobin (HbR) Concentration Between the Emotion and Non-Emotion Conditions Over the Time Courses of the Semantic Fluency Tests. Red and blue lines indicate differences in HbO and HbR, respectively. The verbal fluency task started at *t* = 0 s and ended at *t* = 60 s. Error bars denote one standard error ± the mean difference.

Next, linear mixed model analyses were conducted to investigate the effects of condition, hemisphere, and region on mean changes in HbO and HbR during the SFTs. The full results are presented in Table 3, and the differences in mean changes in HbO and HbR between the two SFTs as time unfolded are illustrated in Fig. 3. For HbO, a significant effect of hemisphere was found, *F*(1, 326) = 9.11, *p* = 0.003, owing to a significantly larger mean increase in HbO in the left than the right hemisphere overall. The effect of region was also significant. Post-hoc tests with the Sidak correction showed that the ventrolateral PFC exhibited a significantly greater mean increase in HbO than the frontopolar or dorsolateral PFC regions. These two latter regions also displayed significantly greater mean increases in HbO compared with the dorsomedial and posterolateral frontal regions. No other main or interaction effects, including the main effect of condition, were significant, *F*s < 3.58, *p*s > 0.060.

**Table 3:** Linear Mixed Model Results for Mean Changes in Frontal Oxyhemoglobin (ΔHbO) and Deoxyhemoglobin (ΔHbR) Concentration During the Semantic Fluency Tests.

	*F*	*df*	*p*	Comparison (with Sidak correction)
Δ**HbO**				
Condition (non-emotion, emotion)	3.58	1, 228	.060	–
Hemisphere (left, right)	9.11	1, 326	.003**	Left > Right
Region (frontopolar, dorsomedial, dorsolateral, ventrolateral, posterolateral)	53.91	4, 585	< .001***	Ventrolateral > frontopolar, dorsolateral > dorsomedial, posterolateral
Condition × hemisphere	0.00	1, 270	.96	–
Condition × region	0.19	4, 587	.95	–
Hemisphere × region	0.07	4, 565	.99	–
Condition × hemisphere × region	0.12	4, 580	.97	–
Δ**HbR**				
Condition (non-emotion, emotion)	0.50	1, 285	.48	–
Hemisphere (left, right)	4.90	1, 401	.027*	Left < right
Region (frontopolar, dorsomedial, dorsolateral, ventrolateral, posterolateral)	38.30	4, 605	< .001***	Ventrolateral < frontopolar, dorsolateral < dorsomedial, posterolateral
Condition × hemisphere	0.75	1, 336	.39	–
Condition × region	1.13	4, 612	.34	–
Hemisphere × region	0.26	4, 600	.90	–
Condition × hemisphere × region	0.01	4, 610	1.00	–

The formulas for the two linear mixed models applied were ΔHbO (or ΔHbR) ~ condition + hemisphere + region + condition × hemisphere + condition × region + hemisphere × region + condition × hemisphere × region + (1 | subject).

Similarly, for HbR, a significant effect of hemisphere was observed, which was attributable to a greater mean decrease in HbR in the left than the right hemisphere. In addition, there was a significant effect of region. Post-hoc tests revealed that the mean decrease in HbR in the ventrolateral PFC was significantly greater than that in the frontopolar and dorsolateral PFC regions. The decreases in these two latter regions were also significantly greater than those in the dorsomedial and posterolateral frontal regions. No other main or interaction effects, including the main effect of condition, were significant, *F*s < 1.13, *p*s > 0.060.

### Bayes factors for the differences in frontal cortical activation between conditions

Next, the Bayes factors representing the differences in mean HbO and HbR changes between the two SFT conditions were calculated to evaluate the strength of evidence in favor of the null vs. alternative hypothesis. The Bayes factors were calculated for each channel cluster and are shown in Table 4. For HbO, the Bayes factors ranged from 1.14 to 8.29, eight of which fell between 3 and 10. For HbR, the Bayes factors ranged from 3.00 to 8.36. Taken together, Bayesian statistics suggested that there was substantial evidence in favor of the null hypothesis across chromophores.

**Table 4:** The Bayes Factors for the Differences in Mean Changes in Frontal Oxyhemoglobin (ΔHbO) and Deoxyhemoglobin (ΔHbR) Concentration Between the Emotional and Non-Emotional Semantic Fluency Tests.

	Emotion > non-emotion			
	ΔHbO (μM)		ΔHbR (μM)	
	Mean (*SD*)	Bayes factor	Mean (*SD*)	Bayes factor
**Left**				
Frontopolar	− 0.045 (0.298)	5.23	0.013 (0.113)	6.48
Dorsomedial	− 0.025 (0.223)	6.43	0.009 (0.126)	7.52
Dorsolateral	− 0.062 (0.198)	1.14	0.005 (0.127)	8.11
Ventrolateral	− 0.056 (0.487)	6.39	− 0.04 (0.288)	5.67
Posterolateral	− 0.044 (0.237)	4.12	0.021 (0.14)	5.22
**Right**				
Frontopolar	− 0.009 (0.342)	8.29	− 0.002 (0.114)	8.33
Dorsomedial	− 0.021 (0.289)	7.55	− 0.012 (0.124)	6.87
Dorsolateral	− 0.037 (0.239)	5.15	− 0.017 (0.129)	5.87
Ventrolateral	− 0.092 (0.528)	4.50	− 0.056 (0.25)	3.00
Posterolateral	− 0.085 (0.292)	1.48	− 0.002 (0.156)	8.36

Bayes factor: Null vs. alternative hypothesis.

### Construct and discriminant validity

Lastly, correlation analyses were conducted to validate the emotional SFT and investigate the role of negative mood symptoms in the production of emotion words. The specificity of these relationships was also evaluated by comparing these correlations with those observed for the non-emotion condition. After removing one bivariate outlier with a Cook’s distance greater than 0.5, emotional SFT performance was found to positively correlate with animal fluency performance, *r*(40) = 0.38, *p* = 0.015. This correlation did not significantly differ from the correlation between performance in the non-emotion condition and animal fluency performance [*r*(40) = 0.55], *Z* = 1.32, *p* = 0.19. In addition, based on the entire sample (*n* = 43; i.e., no bivariate outlier), a positive correlation between emotional SFT performance and the DASS-21 total score was identified, *r*(41) = 0.31, *p* = 0.046. More importantly, this correlation was significantly larger than the correlation between performance on the non-emotional SFT and the DASS-21 total score [*r*(41) = − 0.081], *Z* = 2.55, *p* = 0.011.

## Discussion

There has been growing interest in combining the verbal fluency test with fNIRS to study brain functioning and to screen for neuropsychiatric disorders (see^9^, for a systematic review and meta-analysis). In light of the difference between emotion and neutral word processing (e.g.,^16^), an emotional analogue of the SFT has been recently developed^19^. However, the neural underpinnings of the emotional SFT remain elusive, limiting the full use of this new test and knowledge about this test’s contribution to the verbal fluency test–fNIRS literature. The present study aimed to bridge this knowledge gap by using fNIRS to examine frontal lobe processing during the emotional SFT. The results showed that the emotional SFT, similar to the non-emotional SFT, elicited significant activation in the bilateral frontopolar, dorsolateral, and ventrolateral PFC. There was no significant difference in frontal cortical activation between the two test versions. These null differences occurred in a context where non-emotional and emotional SFT performances showed differential relationships with self-reported negative mood symptoms.

In keeping with previous findings with other languages^19,25^, the participants generated significantly fewer Chinese words on the emotional than the non-emotional SFT. As emotional SFT performance has been shown to be associated with sympathetic activation^19^, the slower retrieval of emotion words compared with neutral words may be related to its reliance on the experience and embodiment of emotions^58^. In addition, emotional SFT performance significantly positively correlated with both animal fluency performance and the overall level of negative mood symptoms. Notably, the relationship between word production and mood symptoms was specific to the emotional SFT, which could be related to the enhanced effect of negative mood on processing and retrieving emotional information^18,59^. This finding of a specific relationship elaborates the previously reported positive correlation between emotional SFT performance and the DASS total score^19^ (but see^20^), providing evidence for discriminant validity for the emotional SFT.

Both the HbO and HbR data suggest that multiple PFC regions were activated during the SFT, regardless of the test version. Pronounced activation was found in the ventrolateral PFC, which is consistent with the fMRI and fNIRS literature and the putative role of the (left) ventrolateral PFC in the controlled access to long-term memory^26^ and inhibition of dominant responses^27^. There was also activation, albeit to a lesser extent, in the frontopolar cortex and dorsolateral PFC. These findings are consistent with the role of the frontopolar cortex in the integration and coordination of information among various PFC regions^33,34^, and the involvement of the dorsolateral PFC in shifting attention between sets^28^ and monitoring working memory^29,30^. Moreover, the stronger activation in the left PFC than in the right PFC corroborates the dominant role of the left hemisphere in verbal production^60^. The simultaneous increase in HbO and decrease in HbR, along with the high degree of the regional specificity of changes, provide compelling evidence for the existence of neural activity and not just systemic changes.

There were no significant differences in activation in terms of changes in HbO or HbR between the non-emotional and emotional SFTs. Bayesian analyses performed with the two chromophores also yielded converging, substantial evidence supporting the null hypothesis (i.e., no difference between conditions) across frontal cortical regions. Previous studies have shown that emotional SFT performance was associated with sympathetic activation^19^, and that the regulation of autonomic arousal states was mediated by limbic regions (e.g., the amygdala), anterior cingulate cortex, and medial PFC^31,61^. According to Etkin et al.^31^, activation foci associated with sympathetic activity are located along the anterior cingulate gyrus. Therefore, the present lack of activation difference between the non-emotional and emotional SFTs might be due to the lack of sensitivity for fNIRS to detect activation in deep brain regions. Studies using fMRI to compare activation across the whole brain between the two SFT conditions would help to address this issue. Alternatively, the null results could reflect the comparable involvement of frontal lobe subregions during emotional and non-emotional cognitive control tasks, as demonstrated in the domain of inhibitory control^62,63^.

The present study involved the application of the conventional verbal fluency test–fNIRS method, with the novelty of comparing neural processing during the emotional and non-emotional SFTs. That is, the present probe locations, which covered the bilateral frontopolar, ventrolateral, and dorsolateral PFC, as well as the paradigm design that involved alternations between a control task and the verbal fluency test, were comparable to those employed in previous studies (see^9^ for the methods used in 121 verbal fluency test–fNIRS studies in psychiatric disorders). Therefore, the present findings do not lend support to the unique utility of integrating the emotional SFT with the current verbal fluency test-fNIRS method. However, since the present study involved only healthy young adults, the findings of this study may not be generalizable to other age groups or clinical populations. It would be beneficial for other studies to retest activation differences in frontal cortex and other brain regions during the non-emotional and emotional SFTs in other populations to expand the present findings.

The present findings provide no clue as to whether the same frontal cortical regions are necessary for non-emotional and emotional SFT performances. For phonemic and (non-emotional) semantic fluency, neuropsychological and neuroimaging studies to date have yielded inconsistent evidence regarding differences in the neural bases of these two verbal fluency types. Specifically, lesion studies have implicated overlapping yet distinct brain regions and white matter tracts in phonemic and semantic fluency^64–66^. In contrast, an fMRI meta-analysis has failed to identify significant differences in activation between the two versions^12^; fNIRS studies have also reported mixed results [e.g., ^15,67^]. Thus, even though the two verbal fluency tasks may activate similar brain regions, it is still possible that different regions contribute to the two tasks. The same also applies to the current study, and whether different brain regions contribute to non-emotional and emotional SFT performances requires further investigations.

In summary, the present study has clarified neural processing during the emotional SFT. Converging evidence from changes in HbO and HbR suggests that the emotional SFT engages various frontal cortical regions in a comparable way to the conventional, non-emotional SFT. Despite this similarity, there is evidence for discriminant validity for the emotional SFT because performance on this test is specifically related to self-reported negative mood symptoms. Therefore, future work would benefit from conducting a comprehensive neurophysiological assessment, such as combined fNIRS or fMRI and peripheral physiological measurements^68,69^, to better understand the contributions of the central and autonomic nervous systems, and the interactions between the two systems, while performing the emotional SFT. Studies that apply the emotional SFT to populations associated with both semantic fluency deficits and difficulty in recognizing and verbalizing one’s own emotional experiences, including depression^7,22^ and schizophrenia^5,23^, will also facilitate understanding of the clinical utility of this test.
